# Supplementary material for: Trends in Molecular Diagnosis and Diversity Studies for Phytosanitary Regulated Xanthomonas
Source: Microorganisms. 2021 Apr 16;9(4):862. doi: 10.3390/microorganisms9040862 (PMC8073235; doi:10.3390/microorganisms9040862)
Supplement: Supplementary file 1 [file microorganisms-09-00862-s001.zip › microorganisms-1186978-Supplementary Table 2.pdf]

microorganisms

MDPI

Article supplementary materials

Supplementary materials belonging to

Trends In Molecular Diagnosis And Diversity Studies For Phytosanitary Regulated *Xanthomonas*

Vittoria Catara, Jaime Cubero, Joël F. Pothier, Eran Bosis, Claude Bragard, Edyta Đermić, Maria C. Holeva, Marie-Agnès Jacques, Francoise Petter, Olivier Pruvost, Isabelle Robène, David J. Studholme, Fernando Tavares, Joana G. Vicente, Ralf Koebnik and Joana Costa

Supplementary Table 2: Characteristics of MLVA schemes developed for *Xanthomonas* spp.

Target	Number of TR loci	Range of repeat unit sizes (bp)	Reference
X. albilineans	15	3-36	[1]
X. arboricola	26	7-20	[2]
X. arboricola pv. *pruni*	6	6-20	[3]
X. arboricola pv. *pruni*	23	6-116	[4]
X. citri pv. *citri*	14	6-7	[5]
X. citri pv. *citri*	31	10-217	[6]
X. citri pv. *mangiferaeindicae*	12	6-7	[7]
X. citri pv. *viticola*	8	7	[8]
X. fragariae	36	3-33	[9]
X. euvesicatoria	16	6-8	[10]
X. oryzae	16	6-12	[11]
X. oryzae pv. *oryzicola*	25	5-9	[12]
X. phaseoli pv. *manihotis*	22	6-26	[13]
X. phaseoli pv. *manihotis*	15	6-7	[14]
X. vasicola pv. *musacearum*	19	6-12	[15]

Genotyping schemes analysing a relatively large number of TR loci are desirable as they allow to partially control their homoplastic evolution [16].

References

1. Tardiani, A.C.; Perecin, D.; Peixoto-Junior, R.F.; Sanguino, A.; Landell, M.M.G.; Beriam, L.O.; Nunes Daniel, S.; Camargo, L.E.A.; Creste, S. Molecular and pathogenic diversity among Brazilian isolates of *Xanthomonas albilineans* assessed with SSR marker loci. *Plant Dis.* **2013**, *98*, 540–546, doi:10.1094/PDIS-07-13-0762-RE.
2. Cesbron, S.; Pothier, J.; Gironde, S.; Jacques, M.A.; Manceau, C. Development of multilocus variable-number tandem

repeat analysis (MLVA) for *Xanthomonas arboricola* pathovars. *J. Microbiol. Methods* **2014**, *100*, 84–90, doi:10.1016/j.mimet.2014.02.017.

3. Bergsma-Vlami, M.; Martin, W.; Koenraadt, H.; Teunissen, H.; Pothier, J.F.; Duffy, B.; Van Doorn, J. Molecular typing of Dutch isolates of *Xanthomonas arboricola* pv. *pruni* isolated from ornamental cherry laurel. *J. Plant Pathol*. **2012**, *94*, S1.29–S1.35, doi:10.4454/jpp.v94i1sup.006.
4. López-Soriano, P.; Boyer, K.; Cesbron, S.; Morente, M.C.; Peñalver, J.; Palacio-Bielsa, A.; Vernière, C.; Ló Pez, M.M.; Pruvost, O.; Vinatzer, B.A.; et al. Multilocus variable number of tandem repeat analysis reveals multiple introductions in Spain of *Xanthomonas arboricola* pv. *pruni*, the causal agent of bacterial spot disease of stone fruits and almond. *PLoS ONE* **2016**, *11*, e0163729, doi:10.1371/journal.pone.0163729.
5. Ngoc, L.B.T.; Verniere, C.; Vital, K.; Guerin, F.; Gagnevin, L.; Brisse, S.; Ah-You, N.; Pruvost, O. Development of 14 minisatellite markers for the citrus canker bacterium, *Xanthomonas citri* pv. *citri*. *Mol. Ecol. Resour.* **2009**, *9*, 125–127, doi:10.1111/j.1755-0998.2008.02242.x.
6. Pruvost, O.; Magne, M.; Boyer, K.; Leduc, A.; Tourterel, C.; Drevet, C.; Ravigné, V.; Gagnevin, L.; Guérin, F.; Chiroleu, F.; et al. A MLVA genotyping scheme for global surveillance of the citrus pathogen *Xanthomonas citri* pv. *citri* suggests a worldwide geographical expansion of a single genetic lineage. *PLoS ONE* **2014**, *9*, e98129, doi:10.1371/journal.pone.0098129.
7. Pruvost, O.; Vernière, C.; Vital, K.; Guérin, F.; Jouen, E.; Chiroleu, F.; Ah-You N.; Gagnevin, L. Insertion sequence- and tandem repeat-based genotyping techniques for *Xanthomonas citri* pv. *mangiferaeindicae*. *Phytopathology* **2011**, *101*, 887–893, doi:10.1094/PHYTO-11-10-0304.
8. Ferreira, M.A.S.V.; Bonneau, S.; Briand, M.; Cesbron, S.; Portier, P.; Darrasse, A.; Gama, M.A.S.; Barbosa, M.A.G.; Mariano, L.R.; de Souza, E.B.; et al. *Xanthomonas citri* pv. *viticola* affecting grapevine in Brazil: Emergence of a successful monomorphic pathogen. *Front. Plant Sci.* **2019**, *10*, 489, doi:10.3389/fpls.2019.00489.
9. Gétaz, M.; Krijger, M.; Rezzonico, F.; Smits, T.H.M.; van der Wolf, J.M.; Pothier, J.F. Genome-based population structure analysis of the strawberry plant pathogen *Xanthomonas fragariae* reveals two distinct groups that evolved independently before its species description. *Microb. Genom.* **2018**, *4*, e000189, doi:10.1099/mgen.0.000189.
10. Vancheva, T.; Bogatzevska, N.; Moncheva, P.; Mitrev, S.; Vernière, C.; Koebnik, R. Molecular epidemiology of *Xanthomonas euvesicatoria* strains from the Balkan Peninsula revealed by a new Multiple-Locus Variable-Number Tandem-Repeat Analysis scheme. *Microorganisms* **2021**, *9*, 536, doi:10.3390/microorganisms9030536.
11. Poulin, L.; Grygiel, P.; Magne, M.; Gagnevin, L.; Rodriguez-R, L.M.; Forero Serna, N.; Zhao, S.; El Rafii, M.; Dao, S.; Tekete, C.; et al. New multilocus variable-number tandem-repeat analysis tool for surveillance and local epidemiology of bacterial leaf blight and bacterial leaf streak of rice caused by *Xanthomonas oryzae*. *Appl. Environ. Microbiol.* **2015**, *81*, 688–698, doi:10.1128/AEM.02768-14.
12. Zhao, S.; Poulin, L.; Rodriguez-R, L.M.; Forero Serna, N.; Liu, S.Y.; Wonni, I.; Szurek, B.; Verdier, V.; Leach, J.E.; He, Y.Q.; et al. Development of a variable number of tandem repeats typing scheme for the bacterial rice pathogen *Xanthomonas oryzae* pv. *oryzicola*. *Phytopathology* **2012**, *102*, 948–956, doi:10.1094/PHYTO-04-12-0078-R.
13. Arrieta-Ortiz, M.L.; Rodríguez, R.L.M.; Pérez-Quintero, Á.L.; Poulin, L.; Díaz, A.C.; Rojas, N.A.; Trujillo, C.; Benavides, M.R.; Bart, R.; Boch, J.; et al. Genomic survey of pathogenicity determinants and VNTR markers in the cassava bacterial pathogen *Xanthomonas axonopodis* pv. *manihotis* strain CIO151. *PLoS ONE* **2013**, *8*, e79704, doi:10.1371/journal.pone.0079704.
14. Rache, L.; Blondin, L.; Flores, C.; Trujillo, C.; Szurek, B.; Restrepo, S.; Koebnik, R.; Bernal, A.; Vernière, C. An Optimized microsatellite scheme for assessing populations of *Xanthomonas phaseoli* pv. *manihotis*. *Phytopathology* **2019**, *109*, 859–869, doi:10.1094/PHYTO-06-18-0210-R.
15. Nakato, G.V.; Fuentes Rojas, J.L.; Verniere, C.; Blondin, L.; Coutinho, T.; Mahuku, G.; Wicker, E. A new Multi Locus Variable Number of Tandem Repeat Analysis scheme for epidemiological surveillance of *Xanthomonas vasicola* pv. *musacearum*, the plant pathogen causing bacterial wilt on banana and enset. *PLoS ONE* **2019**, *14*, e0215090, doi:10.1371/journal.pone.0215090.
16. Estoup, A.; Jarne, P.; Cornuet, J.M. Homoplasy and mutation model at microsatellite loci and their consequences for population genetics analysis. *Mol. Ecol*. **2002**, *11*, 1591–1604, doi:10.1046/j.1365-294x.2002.01576.x.
