# Supplementary material for: Trends in Molecular Diagnosis and Diversity Studies for Phytosanitary Regulated Xanthomonas
Source: Microorganisms. 2021 Apr 16;9(4):862. doi: 10.3390/microorganisms9040862 (PMC8073235; doi:10.3390/microorganisms9040862)
Supplement: Supplementary file 1 [file microorganisms-09-00862-s001.zip › microorganisms-1186978-Supplementary Table 1.pdf]

microorganisms

MDPI

Article supplementary materials

Supplementary materials belonging to

Trends In Molecular Diagnosis And Diversity Studies For Phytosanitary Regulated *Xanthomonas*

Vittoria Catara, Jaime Cubero, Joël F. Pothier, Eran Bosis, Claude Bragard, Edyta Đermić, Maria C. Holeva, Marie-Agnès Jacques, Francoise Petter, Olivier Pruvost, Isabelle Robène, David J. Studholme, Fernando Tavares, Joana G. Vicente, Ralf Koebnik and Joana Costa

Supplementary Table S1 Short description of detection and identification methods included in official protocols on regulated phytopathogenic *Xanthomonas* species[1,2,3]

Pathogen	Purpose of method	Plant part tested	Isolation	Serological methods	Molecular methods	Other methods
X. euvesicatoria pv. *allii* [1]	Screening	Symptomatic: Plant material	Direct isolation (non-selective media: YPGA; selective media: NCTM1, KC, modified MXP)		●Duplex nested-PCR [2] and RFLP with CfrI (EaeI) and NheI<br>●Triplex real-time PCR [3]	
		Asymptomatic: Seeds	Direct isolation (semi-selective medium: NCTM1, KC, modified MXP)		●Duplex nested PCR [2] and RFLP with CfrI (EaeI) and NheI<br>●Triplex real-time PCR [3]	
	Identification		Colony morphology		●First step of duplex nested-PCR [2], and RFLP (as for screening) if necessary.<br>●Triplex real-time PCR [3]<br>●Rep-PCR [4–6]<br>●AFLP [7–9]<br>●DNA sequencing (*gyrB*, *atpD*, *dnaK*, *efp*) [7,10]	●Biochemical, physiological tests<br>●Automated Biolog identification system<br>●Pathogenicity test on susceptible cultivar of *Allium cepa* (e.g. Red Creole): plants or detached leaf test
X. arboricola pv. *corylina* [11]	Screening	Symptomatic: Plant material	Direct isolation (non-selective media: GYCA, YPGA)			
		Asymptomatic: no standardized methods for plant material, but buds would be the best candidate organs	As with symptomatic tissue			
	Identification		Colony morphology			●Biochemical tests<br>●Hypersensitive response on tobacco<br>●Pathogenicity test: inoculation of: a) buds, or b) twigs
X. phaseoli pv. *dieffenbachiae* [12][3]	Screening	Symptomatic: Plant material	Direct isolation (non-selective medium: YPGA; semi-selective media: NCTM4, CS, modified ET)	●IF<br>●DAS-ELISA	Nested-PCR [13]	
		Asymptomatic: Plant material	As with symptomatic tissues with enrichment using ET medium.	●IF<br>●DAS-ELISA<br>(both methods using enriched	Nested-PCR [13] (using enriched extracts in ET-medium)	

Pathogen	Purpose of method	Plant part tested	Isolation	Serological methods	Molecular methods	Other methods
				extracts in ET-medium)		
	Identification		Colony morphology	•IF<br>•Indirect-ELISA	•First step of nested-PCR [13] followed by RFLP with HincII<br>•Sequencing single genes (e.g. *gyrB*) [10,14]<br>•DNA barcoding (16S rDNA; *gyrB*; *avrBs2*)[15]	•Catabolic biochemical tests;<br>•Hypersensitive response on tobacco<br>•Pathogenicity test on *Anthurium* plants (leaves or stems)
Xanthomonas spp. on tomato and sweet pepper) [16][3]	Screening	Symptomatic: Plant material	Direct isolation (non-selective medium: Wilbrink's medium, NSCAA, YPGA)	•IF<br>•ELISA	•Conventional PCR specific for *X. euvesicatoria* [17]<br>•Conventional PCR of unknown specificity [18]	
		Asymptomatic: seeds	Direct isolation (non-selective media: YGCA, Wilbrink's medium, NA, ND, YDC, NBY, adenine supplemented YPGA; semi-selective media: CKTM, mMXV, mTMB)	•IF<br>•ELISA	As with symptomatic tissues	
	Identification		Colony morphology	•IF<br>•ELISA	•Duplex-PCR [19]: two separate reactions, each including three primer combinations.<br>•Rep-PCR fingerprinting [20]<br>•DNA barcoding (16S rDNA; *gyrB*; *avrBs2*); for *X. euvesicatoria* and *X. perforans* all three genes, for *X. vesicatoria* and *X. gardnerii* only the first two genes [15]	•Biochemical tests (especially assays for amylolytic and pectolytic activity)<br>•Protein profile (SDS-PAGE)<br>•Automated Biolog identification system<br>•Fatty acid methyl ester analysis (FAME)<br>•Hypersensitive response on bean pods<br>•Pathogenicity test on tomato or pepper plants (e.g. tomato cv. Moneymaker; pepper cv. Early Calwonder)
Xanthomonas spp. on tomato and sweet pepper [21,22][2,3]	Screening	Asymptomatic: seeds	Direct isolation (semi-selective media: mTMB, CKTM)			
	Identification		Colony morphology (semi-selective media: mTMB, CKTM; non-selective medium: YDC medium)		Real-time PCR:<br>a) AFLP derived Taqman® PCR, and<br>b) XopD Taqman® PCR<br>(both real-time PCRs are needed as they are considered 'complementary for some isolates and together cover the entire tomato and pepper *Xanthomonas* bacterial spot collection that was tested in validation studies)	Pathogenicity test on tomato or pepper (e.g. tomato cv. Cal Ace; pepper cv. Early Cal Wonder)
X. arboricola pv. *pruni* [23]	Screening	Symptomatic: plant material (leaves, immature fruits; not possible from ripening fruits)	Direct isolation (non-selective media: Wilbrink's medium, YPG, GYCA, or YDC)		•Conventional PCR [24]<br>•Real-time PCR [25]<br>•LAMP [26]	
		Asymptomatic: dormant scion chips, one-year twigs<br>Note: methods validated on peach and plum, but not on cherry, almond, apricot.	Direct isolation (non-selective media: Wilbrink's medium, YPGA, GYCA or YDC)		•Conventional PCR [24]<br>•Real-time PCR [25]<br>•LAMP [26]	

microorganisms

MDPI

Pathogen	Purpose of method	Plant part tested	Isolation	Serological methods	Molecular methods	Other methods
	Identification		Colony morphology	IF	●Real-time PCR [25]<br>●Real-time PCR [27]<br>●LAMP [26]<br>●Rep-PCR fingerprinting [20]<br>●DNA barcoding (additional info to support identification)	●Biochemical tests<br>●HR on leaves tobacco (e.g., cvs 'Samsun' or 'Xanthi') or tomato (e.g., cvs 'Moneymaker' or 'Roma')<br>●Protein profile (SDS-PAGE)<br>●Fatty acids methyl-ester profile analysis (FAME)<br>●Pathogenicity test on susceptible peach or plum: a) detached leaf inoculation (e.g. seedlings cv. 'Sunhigh', cherry laurel cv. 'Novita' or cv. 'Rotundifolia' or any other cultivar known to be susceptible to *X. arboricola* pv. *pruni*); b) plant inoculation (peach cvs. 'Barrier', 'Catherine', 'Parade', 'Royal Glory' 'Rich Lady' or Sunhigh; plum cvs. 'Angeleno', 'Black Star', 'Black Amber', 'TC Sun', 'Golden Plum', 'Fortune,' 'Anne Gold')
X. phaseoli pv. phaseoli and *X. citri pv. fuscans* [28–30]	Screening	Asymptomatic:<br>seeds	Direct isolation<br>(semi-selective media: MT, XCP1)			
	Identification		Colony morphology on MT, XCP1; subculture in YDC		●Conventional PCR [31]<br>●Conventional PCR [32]	Pathogenicity test on susceptible (e.g. 'Flavert' or 'Michelet') bean seedlings (dipping leaves in the bacterial suspension)
X. fragariae [33][1]	Screening	Symptomatic:<br>plant material (leaves, crown and petioles)	●Direct isolation (media: Wilbrink's medium with nitrate and YPGA; SPA may be used but not validated; use of purified agar in all media is recommended)<br><br>●Isolation after enrichment via detached leaf assay (medium: Wilbrink's medium with nitrate)	●IF<br>●Indirect ELISA<br>●DAS-ELISA	●Multiplex PCR [34]<br>●Nested PCR (not validated; [35]<br><br>PCR can be performed before or after enrichment (i.e. detached leaf inoculation, leaf tissue maceration and streaking on Wilbrink's medium with nitrate, washing off the bacterial growth from the culture medium and PCR analysis)	Bioassay: Detached strawberry leaf assay directly using the macerated sample for inoculation (susceptible cultivars: e.g. 'Camarosa', 'Seascape', 'Selva', 'Korona')
		Asymptomatic:<br>plant material (runners, crown and petioles)	Direct isolation<br>(as for symptomatic samples)	As for symptomatic samples	As for symptomatic samples	
	Identification		Colony morphology	●IF<br>●Indirect ELISA<br>●DAS-ELISA	●Multiplex PCr [34]<br>●Conventional PCR [36]<br>●Rep-PCR fingerprinting [37]<br>●DNA barcoding (16S rDNA; *gyrB*) [20]	●Biochemical and physiological tests<br>●Profile in API 20 NE and API 50 CH strips<br>●Fatty acid profiling (FAP)<br>●HR reaction in tobacco leaves (cvs. 'Samsun', 'Xanthi')

Pathogen	Purpose of method	Plant part tested	Isolation	Serological methods	Molecular methods	Other methods
						•Pathogenicity test on strawberry (susceptible cultivars: e.g. 'Camarosa', 'Seascape', 'Selva', 'Korona', 'Pájaro'): detached leaf assay or plant inoculation
X. fragariae [38]	Screening	Symptomatic:<br>Plant material (leaves, crown, petioles)	• Direct isolation (media: Wilbrink's medium with nitrate, YPGA, SPA; use of purified agar in all media is recommended)<br><br>• Isolation after selective enrichment (*in planta*), using detached strawberry leaves (medium: Wilbrink's medium with nitrate)	•IF<br>•Indirect ELISA<br>•DAS-ELISA	•Multiplex PCR [39]<br>•Nested PCR [40]<br>•Nested PCR [41]<br>•Nested PCR [42]<br>•Real-time PCR [43]<br>•Real-time PCR [44]<br>•Real-time PCR [45]; suggested as possibly useful for certification)<br>•Real-time PCR [36]; suggested also for latent infections)<br><br>PCR can be performed after enrichment in vitro (i.e. detached leaf inoculation, leaf tissue maceration and streaking on Wilbrink's medium with nitrate, washing off the bacterial growth from the culture medium and PCR analysis)	Detached strawberry leaf assay directly using the macerated sample for inoculation: test to confirm viable *X. fragariae* (susceptible cultivars: e.g. 'Camarosa', 'Seascape', 'Selva', 'Korona', 'Pájaro')
		Asymptomatic<br>Plant material (leaves, crown, petioles)		As for symptomatic material	• Multiplex PCR [46] (detection up to 103 cfu/ml)<br>•Nested PCR [42] (detection up to 18 target cells in plant tissue)<br>•Nested PCR [40]<br>•Nested PCR [41]<br>•Nested PCR [37]<br>•Real-time PCR [47] (potentially useful for asymptomatic)<br>•Real-time PCR [44] (potentially useful for asymptomatic)	
	Identification		Colony morphology	•Indirect IF<br>•Indirect ELISA<br>•DAS-ELISA	•As for screening PCR protocols<br>•Rep-PCR fingerprinting [48,49]<br>•MLSA (not validated; [50–52]	•Biochemical and physiological tests<br>•Profile in API 20 NE and API 50 CH;<br>•Fatty acid methyl esters (FAMEs)<br>•Pathogenicity test on strawberry (susceptible cultivars: e.g. 'Camarosa', 'Seascape', 'Selva', 'Korona', 'Pájaro'): detached leaf assay or plant inoculation<br>•HR reaction in tobacco leaves (cvs. 'Samsun', 'Xanthi')
X. oryzae pv. oryzicola & *X. oryzae pv. oryzae* [53]	Screening	Symptomatic:<br>Plant material	Direct plating (non-selective media: PSA, NBY, GF, NA; semi-selective media: mXOS, Wakimoto's agar*)<br><br>*without ferrous sulfate for *X. o.* pv. *oryzicola*	Indirect-ELISA		
		Asymptomatic:<br>Plant material (leaves; seedlings)			Conventional PCR [54]	

Pathogen	Purpose of method	Plant part tested	Isolation	Serological methods	Molecular methods	Other methods
		Asymptomatic:<br>Seeds	Direct isolation<br>(media: GF, mXOS)		Bio-PCR (enrichment in PSA medium and PCR by [54]	
	Identification		Colony morphology	Indirect-ELISA	•Conventional PCR ([54]; species specific)<br>•Conventional PCR ([55]; differentiation of the two pathovars; not tested in Europe)<br>•Conventional PCR ([56]; identification of pathovar *oryzae*; not tested in Europe)<br>•DNA barcoding (16S rDNA; *gyrB*) (species specific; [15]	•Biochemical and physiological tests<br>•Fatty acid profiles<br>•Phage-typing<br>•Polyacrylamide gel electrophoresis protein fingerprints<br>•Pathogenicity test in rice plants (susceptible cultivars: IR24, IR8, TNI for *X. oryzae* pv. *oryzae*; IR24, IR50 for *X. oryzae* pv. *oryzicola*; European susceptible varieties recommended to be included): inoculation by leaf clipping (for *X. oryzae* pv. *oryzae*) or spray inoculation (for *X. oryzae* pv. *oryzicola*)
X. citri pv. *aurantifolii* & *X. citri* pv. *citri* [57]	Screening	Symptomatic:<br>lesions from fruit/leaf/stem or other suspicious infected plant material	Isolation<br>(non-selective media: NGA, YPGA, medium described by Canteros et al., 1985; semi-selective media: KCB, containing NGA with kasugamycin, cephalexin, Bravo or Daconil)	IF (possible but commercial antibodies not evaluated)<br>ELISA (possible but mostly for identification)	•Conventional PCR [58]<br>•Conventional PCR (target: *pthA* gene; [59])<br>•Conventional PCR (target: ITS; [59])<br>•Conventional PCR [60]<br>•Nested PCR; Immunocapture and nested PCR (followed by colorimetric detection) [61]<br>•Real-time PCR (developed but not compared with conventional or nested PCR)	
		Asymptomatic:<br>plants, plant parts	Isolation is difficult to succeed			
	Identification		Colony morphology	•IF<br>•Indirect-ELISA	•same sets of primers as for screening<br>•RFLP<br>•Rep-PCR fingerprinting (BOX PCR and ERIC PCR) [62]<br>•Real-time PCR (developed using SYBR green dye or Taqman probes)<br>•DNA barcoding (16S rDNA; *gyrB*; avrBs2) [15]	•Biochemical and physiological tests<br>•Fatty acid analysis<br>•Automated Biolog identification system<br>•Pathogenicity test on susceptible cultivars of citrus hosts (e.g. Duncan grapefruit, Valencia sweet orange, Mexican lime): attached leaf assay or detached leaf assay or inoculation of seedlings growing *in vitro* (e.g. seedlings like 'Marsh' grapefruit, 'Parson brown' sweet orange)
X. citri pv. *aurantifolii* & *X. citri* pv. *citri* [63]	Screening	Symptomatic:<br>lesions from fruits/leaves/shoot/twigs, or tissue from cankers on twigs/branches/trunk/collar	Isolation<br>(non-selective media: NGA, YPGA, Wakimoto medium; semi-selective media: KC, KCB)	•IF<br>•DAS-ELISA	•Conventional PCR [61]<br>•Conventional PCR (target: *pthA* gene; [59])<br>•Nested PCR; Immunocapture and nested PCR (followed by colorimetric detection) [61]<br>•Real-time PCR [64]	Bioassay with plant sample extract: a) inoculation test in leaf discs; b) detached leaf enrichment

Pathogen	Purpose of method	Plant part tested	Isolation	Serological methods	Molecular methods	Other methods
		Asymptomatic: plants, plant parts	Isolation (washing off leaves or fruits in peptone buffer and plating; enrichment on semi-selective medium: XOS)	●IF	As for symptomatic material	Bioassay with plant sample extract: a) inoculation test in leaf discs; b) detached leaf enrichment
	Identification		Colony morphology	● IF<br>● DAS-ELISA<br>● Indirect-ELISA	●Conventional PCR [57]<br>●Conventional PCR (target: *pthA* gene; [58])<br>●Conventional PCR (target: ITS; [58])<br>●Conventional PCR [65]<br>●Conventional PCR [66]<br>●Sequencing PCR amplicons<br>●DNA-DNA hybridization [67]<br>●Genomic fingerprinting [68,69]<br>●Rep-PCR fingerprinting (BOX PCR and ERIC PCR) [4,58]<br>●MLSA [7,51,70]	●Biochemical and physiological tests<br>●Bioassay of leaf discs or detached leaves<br>●Pathogenicity test on susceptible cultivars of citrus hosts (e.g. Duncan grapefruit, Valencia sweet orange, Mexican lime): leaf assays (infiltration)

Notes:

1. Protocol under revision or revision planned.
2. Official protocols can be sourced from the open-access websites of these organizations, e.g. European and Mediterranean Plant Protection Organization (EPPO), Food and Agricultural Organization of the United Nations (FAO) via IPPC, International Seed Testing Association (ISTA), International Seed Federation (ISF), USDA National Seed Health System (NSHS). Other sources of reviewed detection methods include scientific publications of EFSA ('Scientific Opinions', 'Pest Cards', 'Pest categorisation', etc).
3. No official methods are available for: *Xanthomonas axonopodis* pv. *poinsettiicola, X. arboricola* pv. *juglandis, X. arboricola* pv. *fici* and *X. translucens* pv. *translucens*. For *X. translucens* and *X. fuscans* subsp. *fuscans,* an identification method based on DNA barcoding is described in [15].

References:

1. European and Mediterranean Plant Protection Organization (EPPO). *Xanthomonas axonopodis* pv. *allii*. *EPPO Bull.* **2016**, *46*, 429–443, doi:10.1111/epp.12329.
2. Robène-Soustrade, I.; Legrand, D.; Gagnevin, L.; Chiroleu, F.; Laurent, A.; Pruvost, O. Multiplex nested PCR for detection of *Xanthomonas axonopodis* pv. *allii* from onion seeds. *Appl. Environ. Microbiol.* **2010**, *76*, 2697–2703, doi:10.1128/AEM.02697-09.
3. Robène, I.; Perret, M.; Jouen, E.; Escalon, A.; Maillot, M.V.; Chabirand, A.; Moreau, A.; Laurent, A.; Chiroleu, F.; Pruvost, O. Development and validation of a real-time quantitative PCR assay to detect *Xanthomonas axonopodis* pv. *allii* from onion seed. *J. Microbiol. Methods* **2015**, *114*, 78–86, doi:10.1016/j.mimet.2015.04.017.
4. Alvarez, A.M.; Benedict, A. A.; Mizumoto, C. Y.; Hunter, J. E.; Gabriel, D. W. Serological, pathological, and genetic diversity among strains of *Xanthomonas campestris* infecting Crucifers. *Phytopathology* **1994**, *84*, 1449–1457, doi:10.1094/Phyto-84-1449.
5. Gent, D.H.; Schwartz, H.F.; Ishimaru, C.A.; Louws, F.J.; Cramer, R.A.; Lawrence, C.B. Polyphasic characterization of *Xanthomonas* strains from onion. *Phytopathology* **2004**, *94*, 184–195, doi:10.1094/PHYTO.2004.94.2.184.
6. Gent, D.H.; Al-Saadi, A.; Gabriel, D.W.; Louws, F.J.; Ishimaru, C.A.; Schwartz, H.F. Pathogenic and genetic relatedness among *Xanthomonas axonopodis* pv. *allii* and other pathovars of *X. axonopodis*. *Phytopathology* **2005**, *95*, 918–925, doi:10.1094/PHYTO-95-0918.
7. Ngoc, L.B.T.; Vernière, C.; Jouen, E.; Ah-You, N.; Lefeuvre, P.; Chiroleu, F.; Gagnevin, L.; Pruvost, O. Amplified fragment length polymorphism and multilocus sequence analysis-based genotypic relatedness among pathogenic variants of *Xanthomonas citri* pv. *citri* and *Xanthomonas campestris* pv. *bilvae*. *Int. J. Syst. Evol. Microbiol.* **2010**, *60*, 515–525, doi:10.1099/ijs.0.009514-0.
8. Vos, P.; Hogers, R.; Bleeker, M.; Reijans, M.; van de Lee, T.; Hornes, M.; Frijters, A.; Pot, J.; Peleman, J.; Kuiper, M.; et al. AFLP: A new technique for DNA fingerprinting. Nucleic Acids Res. 1995, 23, 4407–4414, doi:10.1093/nar/23.21.4407.
9. Roumagnac, P.; Gagnevin, L.; Gardan, L.; Sutra, L.; Manceau, C.; Dickstein, E.R. Polyphasic characterization of xanthomonads isolated from onion, garlic and Welsh onion (*Allium* spp.) and their relatedness to different *Xanthomonas* species. *Int. J. Syst. Evol. Microbiol.* **2004**, *54*, 15–24, doi:10.1099/ijs.0.02714-0.
10. Parkinson, N.; Cowie, C.; Heeney, J.; Stead, D. Phylogenetic structure of *Xanthomonas* determined by comparison of *gyrB* sequences. *Int. J. Syst. Evol. Microbiol.* **2009**, *59*, 264–274, doi:10.1099/ijs.0.65825-0.
11. European and Mediterranean Plant Protection Organization (EPPO). *Xanthomonas arboricola* pv. *corylina*. *EPPO Bull.* **2004**, *34*, 179–181, doi:10.1111/j.1365-2338.2004.00716.x.
12. European and Mediterranean Plant Protection Organization (EPPO). *Xanthomonas axonopodis* pv. *dieffenbachiae*. *EPPO Bull.* **2009**, *39*, 393–402, doi:10.1111/j.1365-2338.2009.02327.x.
13. Robène-Soustrade, I.; Laurent, P.; Gagnevin, L.; Jouen, E.; Pruvost, O. Specific detection of *Xanthomonas axonopodis* pv. *dieffenbachiae* in anthurium (*Anthurium andreanum*) tissues by nested PCR. *Appl. Environ. Microbiol.* **2006**, *72*, 1072–1078, doi:10.1128/AEM.72.2.1072-1078.2006.
14. Young, J.M.; Park, D.C.; Shearman, H.M.; Fargier, E. A multilocus sequence analysis of the genus *Xanthomonas*. *Syst. Appl. Microbiol.* **2008**, *31*, 366–377, doi:10.1016/j.syapm.2008.06.004.
15. European and Mediterranean Plant Protection Organization (EPPO). PM 7/129 (1) DNA barcoding as an identification tool for a number of regulated pests. *EPPO Bull.* **2016**, *46*, 501–537, doi:10.1111/epp.12344.
16. European and Mediterranean Plant Protection Organization (EPPO). *Xanthomonas* spp. (*Xanthomonas euvesicatoria, Xanthomonas gardneri, Xanthomonas perforans, Xanthomonas vesicatoria*) causing bacterial spot of tomato and sweet pepper. *EPPO Bull.* **2013**, *43*, 7–20, doi:10.1111/epp.12018.
17. Moretti, C.; Amatulli, M.T.; Buonaurio, R. PCR-based assay for the detection of *Xanthomonas euvesicatoria* causing pepper and tomato bacterial spot. *Lett. Appl. Microbiol.* **2009**, *49*, 466–471, doi:10.1111/j.1472-765X.2009.02690.x.
18. Park, D.S.; Shim, J.K.; Kim, J.S.; Lim, C.K.; Shrestha, R.; Hahn, J.H.; Kim, H.G. Sensitive and specific detection of *Xanthomonas campestris* pv. *vesicatoria* by PCR using pathovar-specific primers based on rhs family gene sequences. *Microbiol. Res.* **2009**, *164*, 36–42, doi:10.1016/j.micres.2006.11.005.
19. Koenraadt, H.; van Betteray, B.; Germain, R.; Hiddink, G.; Jones, J.B.; Oosterhof, J. Development of specific primers for the molecular detection of bacterial spot of pepper and tomato. *Acta Hortic.* **2009**, *808*, 99–102, doi:10.17660/ActaHortic.2009.808.13.
20. European and Mediterranean Plant Protection Organization (EPPO). PM 7/100 (1) Rep-PCR tests for identification of bacteria. *EPPO Bull.* **2010**, *40*, 365–368, doi:10.1111/j.1365-2338.2010.02409.x.
21. International Seed Federation (ISF). Method for the Detection of *Xanthomonas* spp. on Tomato Seed. Version 5. July 2017. Available online: https://www.worldseed.org/wp-content/uploads/2017/07/Tomato_Xanthomonas_spp_July2017.pdf (accessed on 03/03/2021).
22. International Seed Federation (ISF). Method for the detection of *Xanthomonas* spp. on pepper seed. Version 6, July 2017. Available online: https://www.worldseed.org/wp-content/uploads/2017/07/Pepper_Xanthomonas_spp_July2017.pdf (accessed on 03/03/2021).
23. European and Mediterranean Plant Protection Organization (EPPO). *Xanthomonas arboricola* pv. *pruni*. *EPPO Bull.* **2006**, *36*, 129–133, doi:10.1111/j.1365-2338.2006.00925.x.
24. Pothier, J.F.; Pagani, M.C.; Pelludat, C.; Ritchie, D.F.; Duffy, B. A duplex-PCR method for species- and pathovar-level identification and detection of the quarantine plant pathogen *Xanthomonas arboricola* pv. *pruni*. *J. Microbiol Methods.* **2011**, *86*, 16–24, doi:10.1016/j.mimet.2011.03.019.
25. Palacio-Bielsa, A.; Cubero, J.; Cambra, M.A.; Collados, R.; Berruete, I.M.; López, M.M. Development of an efficient real-time quantitative PCR protocol for detection of *Xanthomonas arboricola* pv. *pruni* in *Prunus* species. *Appl. Environ. Microbiol.* **2011**, *77*, 89–97,

doi:10.1128/AEM.01593-10.
26. Bühlmann, A.; Pothier, J.F.; Tomlinson, J.A.; Frey, J.E.; Boonham, N.; Smits, T.H.M.; Duffy, B. Genomics-informed design of loop-mediated isothermal amplification for detection of phytopathogenic *Xanthomonas arboricola* pv. *pruni* at the intraspecific level. *Plant Pathol.* **2013**, *62*, 475–484, doi:10.1111/j.1365-3059.2012.02654.x.
27. Garita-Cambronero, J.; Palacio-Bielsa, A.; López, M.M.; Cubero, J. Pan-genomic analysis permits differentiation of virulent and non-virulent strains of *Xanthomonas arboricola* that cohabit *Prunus* spp. and elucidate bacterial virulence factors. *Front. Microbiol.* **2017**, *8*, 573, doi:10.3389/fmicb.2017.00573.
28. International Rules for Seed Testing. Detection of *Xanthomonas axonopodis* pv. *phaseoli* and *Xanthomonas axonopodis* pv. *phaseoli var. fuscans* in *Phaseolus vulgaris* (bean) seed. In *Chapter 7: Validated Seed Health Testing Methods*; ISTA: Zürich, Switzerland, 2021; pp. 7–21. Available online: https://www.seedtest.org/upload/cms/user/ISTA_SHMethods_2021_7-021_final.pdf (accessed on 03/03/2021).
29. International Seed Federation. Detection of *Xanthomonas axonopodis* pv. *phaseoli* and var. *fuscans* in Bean Seed. Version 4.1. 2019 Developed by ISHI-Veg. Available online: https://www.worldseed.org/wp-content/uploads/2019/11/Bean_Xap_October_2019.pdf (accessed on 03/03/2021).
30. National Seed Health System. *Method: Be 3.1 ISTA Selective media Assay 7-021:ver 3.2. 2021*; Version 1.4 Date 1.1.2021; NSHS: Ames, IA, USA, 2021.
31. Audy, P. A rapid and sensitive PCR-based assay for concurrent detection of bacteria causing common and halo blights in bean seed. *Phytopathology* **1996**, *86*, 361–366, doi:10.1094/Phyto-86-361.
32. Boureau, T.; Kerkoud, M.; Chhel, F.; Hunault, G.; Darrasse, A.; Brin, C.; Durand, K.; Hajri, A.; Poussier, S.; Manceau, C.; et al. A multiplex-PCR assay for identification of the quarantine plant pathogen *Xanthomonas axonopodis* pv. *phaseoli*. *J. Microbiol. Methods* **2013**, *92*, 42–50, doi:10.1016/j.mimet.2012.10.012.
33. European and Mediterranean Plant Protection Organization (EPPO). *Xanthomonas fragariae. EPPO Bull.* **2006**, *36*, 135–144, doi:10.1111/j.1365-2338.2006.00926.x.
34. Roberts, P.D.; Hodge, N.C.; Bouzar, H.; Jones, J.B.; Stall, R.E.; Berger, R.D.; Chase, A.R. Relatedness of strains of *Xanthomonas fragariae* by restriction fragment length polymorphism, DNA-DNA reassociation, and fatty acid analyses. *Appl. Environ. Microbiol.* **1998**, *64*, 3961–3965, doi:10.1128/aem.64.10.3961-3965.1998.
35. Zimmermann, C.; Hinrisch-Berger, C.; Moltmann, E.; Buchenhauer, H. Nested PCR for detection of *Xanthomonas fragariae* in symptomless strawberry plants. *J. Plant Dis. Prot.* **2004**, *111*, 39–51, doi:10.1007/BF03356131.
36. Vermunt, A.; van Beuningen, A. Monitoring van *Xanthomonas fragariae* in de aardbeiteelt en de ontwikkeling van een hygiëneprotocol. Productschap Tuinbouw en Plantum NL. 2008. Available online: http://www.tuinbouw.nl/project/monitoring-xanthomonas-fragariae-aardbeiteelt (accessed on 03/03/2021).
37. Mahuku, G.S.; Goodwin, P.H. Presence of *Xanthomonas fragariae* in symptomless strawberry crowns in Ontario detected using a nested polymerase chain reaction (PCR). *Can. J. Plant Pathol.* **1997**, *19*, 366–370, doi:10.1080/07060669709501061.
38. Food and Agriculture Organization of the United Nations (FAO). ISPM 27 Diagnostic protocols for regulated pests. DP 14: *Xanthomonas fragariae*. In *International Standard for Phytosanitary Measures No. 27*; FAO: Rome, Italy, 2016; 30p.
39. Hartung, J.S.; Pooler, M.R. Immunocapture and multiplexed-PCR assay for *Xanthomonas fragariae*, causal agent of angular leafspot disease. *Int. Soc. Hortic. Sci.* **1997**, *439*, 821–828, doi:10.17660/ActaHortic.1997.439.136.
40. Moltmann, E.; Zimmermann, C. Detection of *Xanthomonas fragariae* in symptomless strawberry plants by nested PCR. *EPPO Bull.* **2005**, *35*, 53–54, doi:10.1111/j.1365-2338.2005.00812.x.
41. Zimmermann, C.; Hinrisch-Berger, C.; Moltmann, E.; Buchenhauer, H. Nested PCR for detection of *Xanthomonas fragariae* in symptomless strawberry plants. *J. Plant Dis. Prot.* **2004**, *111*, 39–51, doi:10.1007/BF03356131.
42. Roberts, P.D.; Jones, J.B.; Chandler, C.K.; Stall, R.E.; Berger, R.D. Survival of *Xanthomonas fragariae* on strawberry in summer nurseries in Florida detected by specific primers and nested polymerase chain reaction. *Plant Dis.* **1996**, *80*, 1283–1288, doi:10.1094/PD-80-1283.
43. Weller, S.A.; Beresford-Jones, N.J.; Hall, J.; Thwaites, R.; Parkinson, N.; Elphinstone, J.G. Detection of Xanthomonas fragariae and presumptive detection of *Xanthomonas arboricola* pv. *fragariae*, from strawberry leaves, by real-time PCR. *J. Microbiol. Methods* **2007**, *70*, 379–383, doi:10.1016/j.mimet.2007.05.018.
44. Vandroemme, J.; Baeyen, S.; Van Vaerenbergh, J.; De Vos, P.; Maes, M. Sensitive real-time PCR detection of *Xanthomonas fragariae* in strawberry plants. *Plant Pathol.* **2008**, *57*, 438–444, doi:10.1111/j.1365-3059.2007.01813.x.
45. Turechek, W.W.; Hartung, J.S.; McCallister, J. Development and optimization of a real-time detection assay for *Xanthomonas fragariae* in strawberry crown tissue with receiver operating characteristic curve analysis. *Phytopathology* **2008**, *98*, 359–368, doi:10.1094/PHYTO-98-3-0359.
46. Ah-You, N.; Gagnevin, L.; Grimont, P.A.D.; Brisse, S.; Nesme, X.; Chiroleu, F.; Bui Thi Ngoc, L.; Jouen, E.; Lefeuvre, P.; Vernière, C.; et al. Polyphasic characterization of *Xanthomonads* pathogenic to members of the *Anacardiaceae* and their relatedness to species of *Xanthomonas*. *Int. J. Syst. Evol. Microbiol.* **2009**, *59*, 306–318, doi:10.1099/ijs.0.65453-0.
47. Ferreira-Tonin, M.; Rodrigues-Neto, J.; Harakava, R.; Lanza Destéfano, S.A. Phylogenetic analysis of *Xanthomonas* based on partial *rpoB* gene sequences and species differentiation by PCR-RFLP. *Int. J. Syst. Evol. Microbiol.* **2012**, *62*, 1419–1424, doi:10.1099/ijs.0.028977-0.
48. Pooler, M.R.; Ritchie, D.F.; Hartung, J.S. Genetic relationships among strains of *Xanthomonas fragariae* based on random amplified polymorphic DNA PCR, repetitive extragenic palindromic PCR, and enterobacterial repetitive intergenic consensus PCR data and generation of multiplexed PCR primers useful for the identification of this phytopathogen. *Appl. Environ. Microbiol.* **1996**, *62*, 3121–3127, doi:10.1128/aem.62.9.3121-3127.1996.
49. Opgenorth, D.C.; Smart, C.D.; Louws, F.J.; Bruijn, F.J.; Kirkpatrick, B.C. Identification of *Xanthomonas fragariae* field isolates by rep-PCR genomic fingerprinting. *Plant Dis.* **1996**, *80*, 868–873, doi:10.1094/PD-80-0868.
50. Parkinson, N.; Aritua, V.; Heeney, J.; Cowie, C.; Bew, J.; Stead, D. Phylogenetic analysis of *Xanthomonas* species by comparison of partial gyrase B gene sequences. *Int. J. Syst. Evol. Microbiol.* **2007**, *57*, 2881–2887, doi:10.1099/ijs.0.65220-0.
51. Almeida, N.F.; Shuangchun, Y.; Rongman, C.; Clarke, C.R.; Morris, C.E.; Schaads, N.W.; Schuenzel, E.L.; Lacy, G.H.; Xiaoan, S.; Jones, J.B.; et al. PAMDB, a multilocus sequence typing and analysis database and website for plant-associated microbes. *Phytopathology* **2010**, *100*, 208–215, doi:10.1094/PHYTO-100-3-0208.
52. Hamza, A.A.; Robene-Soustrade, I.; Jouen, E.; Lefeuvre, P.; Chiroleu, F.; Fisher-Le Saux, M.; Gagnevin, L.; Pruvost, O. MultiLocus sequence analysis and amplified fragment length polymorphism-based characterization of xanthomonads associated with bacterial spot of tomato and pepper and their relatedness to *Xanthomonas* species. *Syst. Appl. Microbiol.* **2012**, *35*, 183–190, doi:10.1016/j.syapm.2011.12.005.
53. European and Mediterranean Plant Protection Organization (EPPO). *Xanthomonas oryzae. EPPO Bull.* **2007**, *37*, 543–553, doi:10.1111/j.1365-2338.2007.01162.x.
54. Sakthivel, N.; Mortensen, C.N.; Mathur, S.B. Detection of *Xanthomonas oryzae* pv. *oryzae* in artificially inoculated and naturally infected rice seeds and plants by molecular techniques. *Appl. Microbiol. Biotechnol.* **2001**, *56*, 435–441, doi:10.1007/s002530100641.
55. Kim, H.M.; Song, W.Y. Characterization of ribosomal RNA intergenic spacer region of several seedborne bacterial pathogens of rice. *Seed Sci. Technol.* **1996**, *24*, 571–580.
56. Adachi, N.; Oku, T. PCR-mediated detection of *Xanthomonas oryzae* pv. *oryzae* by amplification of the 16S-23S rDNA spacer region sequence. *J. Gen. Plant Pathol.* **2000**, *66*, 303–309, doi:10.1007/PL00012969.
57 European and Mediterranean Plant Protection Organization (EPPO). Xanthomonas axonopodis pv. citri. EPPO Bull. 2005, 35, 289–294, doi:10.1111/j.1365-2338.2005.00835.x
58 Hartung, J.S.; Daniel, J.F.; Pruvost, O.P. Detection of Xanthomonas campestris pv. citri by the polymerase chain reaction. Appl. Environ. Microbiol. 1993, 59, 1143–1148
59 Cubero, J.; Graham, J.H. Genetic relationship among worldwide strains of Xanthomonas causing canker in citrus species and design of new primers for their identification by PCR. Appl. Environ. Microbiol. 2002, 68, 1257–1264,
60 Vernière, C.; Hartung, J.S.; Pruvost, O.P.; Civerolo, E.L.; Alvarez, A.M.; Maestri, P.; Luisetti, J. Characterization of phenotypically distinct strains of Xanthomonas axonopodis pv. citri from Southwest Asia. Eur. J. Plant Pathol. 1998, 104, 477–487.
61. Hartung, J. Rapid and sensitive colorimetric detection of Xanthomonas axonopodis pv. citri by immunocapture and a nested-polymerase chain reaction assay. Phytopathology 1996, 86, 95, doi:10.1094/Phyto-86-95.
62. Louws, F.J.; Fulbright, D.W.; Stephens, C.T.; De Bruijn, F.J. Specific genomic fingerprints of phytopathogenic *Xanthomonas* and *Pseudomonas* pathovars and strains generated with repetitive sequences and PCR. *Appl. Environ. Microbiol.* **1994**, *60*, 2286–2295, doi:10.1128/aem.60.7.2286-2295.1994.
63. Food and Agriculture Organization of the United Nations (FAO). ISPM 27 Diagnostic protocols for regulated pests. DP 6: *Xanthomonas citri* subsp. *citri*. In *International Standard for Phytosanitary Measures No. 27*; FAO: Rome, Italy, 2016, 26p.
64. Cubero, J.; Graham, J.H. Quantitative real-time polymerase chain reaction for bacterial enumeration and allelic discrimination to differentiate *Xanthomonas* strains on citrus. *Phytopathology* **2005**, *95*, 1333–1340, doi:10.1094/PHYTO-95-1333.
65. Coletta-Filho, H.D.; Takita, M.A.; De Souza, A.A.; Neto, J.R.; Destéfano, S.A.L.; Hartung, J.S.; Machado, M.A. Primers based on the *rpf* gene region provide improved detection of *Xanthomonas axonopodis* pv. *citri* in naturally and artificially infected citrus plants. *J. Appl. Microbiol.* **2006**, *100*, 279–285, doi:10.1111/j.1365-2672.2005.02787.x.
66. Suk Park, D.; Wook Hyun, J.; Jin Park, Y.; Sun Kim, J.; Wan Kang, H.; Ho Hahn, J.; Joo Go, S. Sensitive and specific detection of *Xanthomonas axonopodis* pv. *citri* by PCR using pathovar specific primers based on *hrpW* gene sequences. *Microbiol. Res.* **2006**, *161*, 145–149, doi:10.1016/j.micres.2005.07.005.
67. Vauterin, L.; Hoste, B.; Kersters, K.; Swings, J. Reclassification of *Xanthomonas*. *Int. J. Syst. Bacteriol.* **1995**, *45*, 472–489, doi:10.1099/00207713-45-3-472.
68. Hartung, J.S.; Civerolo, E.L. Genomic fingerprinting of *Xanthomonas campestris* pv. *citri* strains from Asia, South America and Florida. *Phytopathol.* **1987**, *77*, 282–285, doi:10.1094/Phyto-77-282.

69. Lazo, G.R.; Roffey, R.; Gabriel, D.W. Pathovars of *Xanthomonas campestris* are distinguishable by restriction fragment-length polymorphism. *Int. J. Syst. Evol. Microbiol.* **1987**, *37*, 214–221, doi:10.1099/00207713-37-3-214.
70. Fargier, E.; Le Saux, M.F.; Manceau, C. A multilocus sequence analysis of *Xanthomonas campestris* reveals a complex structure within crucifer-attacking pathovars of this species. *Syst. Appl. Microbiol.* **2011**, *34*, 156–165, doi:10.1016/j.syapm.2010.09.001.
